# Morphometric analysis of aerobic *Eimeria bovis* sporogony using live cell 3D holotomographic microscopy imaging

**DOI:** 10.1007/s00436-021-07338-x

**Published:** 2021-10-11

**Authors:** Sara Lopez-Osorio, Zahady D. Velasquez, Iván Conejeros, Anja Taubert, Carlos Hermosilla

**Affiliations:** 1 CIBAV Research Group, Faculty of Agrarian Sciences, University of Antioquia, Medellín, Colombia; 2 Institute of Parasitology, Biomedical Research Center Seltersberg, Justus Liebig University Giessen, Schuberstrasse 81, 35392 Giessen, Germany

**Keywords:** *Eimeria bovis*, Oocyst, Sporogony, 3D holotomographic microscopy, Live cell imaging

## Abstract

M onoxenous *Eimeria* species are widespread enteropathogenic apicomplexan protozoa with a high economic impact on livestock. In cattle, tenacious oocysts shed by *E. bovis-*infected animals are ubiquitously found and making infection of calves almost inevitable. To become infectious oocysts, exogenous oxygen-dependent *E. bovis* sporogony must occur leading to the formation of sporulated oocysts containing four sporocysts each harboring two sporozoites. Investigations on sporogony by live cell imaging techniques of ruminant *Eimeria* species are still absent in literature as commonly used fluorescent dyes do not penetrate resistant oocyst bi-layered wall. Sporogonial oocysts were daily analyzed by a 3D Cell Explorer Nanolive microscope to explore ongoing aerobic-dependent sporogony as close as possible to an in vivo situation. Subsequently, 3D holotomographic images of sporulating *E. bovis* oocysts were digitally stained based on refractive indices (RI) of oocyst bi-layered wall and sub-compartments of circumplasm using STEVE software (Nanolive), and the cellular morphometric parameters were obtained. Overall, three different *E. bovis* sporogony phases, each of them divided into two sub-phases, were documented: (i) sporoblast/sporont transformation into sporogonial stages, (ii) cytokinesis followed by nuclear division, and finally (iii) formation of four sporocysts with two fully developed sporozoites. Approximately 60% of sporulating *E. bovis* oocysts accomplished aerobic sporogony in a synchronized manner. *E. bovis* sporogony was delayed (i.e., 6 days) when compared to an in vivo situation where 2–3 days are required but under optimal environmental conditions. Live cell 3D holotomography analysis might facilitate the evaluation of either novel disinfectants- or anti-coccidial drug-derived effects on ruminant/avian *Eimeria* sporogony in vitro as discrimination of sporogony degrees based on compactness, and dry mass was here successfully achieved. Main changes were observed in the oocyst area, perimeter, compactness, extent, and granularity suggesting those parameters as an efficient tool for a fast evaluation of the sporulation degree.

## Introduction

The genus *Eimeria* contains apicomplexan enteropathogenic protozoa with a high economic impact on livestock worldwide. The prevalence of *Eimeria* infections in cattle is generally high and might reach 100% in calves (Cornelissen et al. 1995). Despite the dozen *Eimeria* species described to date, *E. bovis* is one of the most pathogenic causing severe typhlocolitis characterized by hemorrhagic diarrhea with sometimes fatal outcome in young animals (Stockdale et al. 1981; Daugschies and Najdrowski 2005). All *Eimeria* species display a monoxenous life cycle composed of two phases, i.e., exogenous and endogenous phases. In the exogenous phase, freshly defecated non-sporulated oocysts are broadly spread into cattle environments; however, they are not still infective until undergoing aerobic sporogony to become infective oocysts. The sporulated oocysts within the genus *Eimeria* contain four sporocysts with two sporozoites in each. The endogenous phase of the life cycle includes numerous asexual merogonies, depending on *Eimeria* species (i.e., *E. bovis* developing two merogonies), in specific host cells, and some specific intestine sections, followed by a sexual gamogony leading after syngamy to the formation of non-sporulated oocysts, which are finally shed through feces into the environment (Hammond et al. 1946; Fayer and Hammond 1967).

As for other species, *E. bovis* oocysts are highly resistant to adverse environmental conditions, such as inadequate moisture, low temperatures, and micro- or anaerobic environments. Optimal conditions for sporogony are temperatures ranging from 15 to 18 °C and sufficient aeration. Additionally, *E. bovis*–sporulated oocysts maintain their infectivity for several months and even survive with ease in harsh Scandinavian or Palearctic winters (Svensson et al. 1994; Lassen et al. 2013). Investigations on *E. bovis* oocyst resistance demonstrated that oocysts incubated in 2% potassium dichromate solution at 4 °C were still infective after 4 and a half years and capable of inducing patent infections of experimentally infected calves (Hermosilla, unpublished data).

There are only a few studies in the literature linking bovine *Eimeria* sporogony with natural oocyst resistance. Accordingly, successful *E. bovis* sporogony seems to result in oocysts capable of overcoming adverse environmental conditions (Svensson et al., 1994). Conversely, closely related *E. zuernii* and *E. alabamensis* oocysts survived much better at sub-zero temperatures as un-sporulated oocyst stages, while *E. bovis* needed to be in a sporulated status to resist very low temperatures (Svensson et al. 1994). Nevertheless, *E. alabamensis*, *E. zuernii*, and *E. ellipsoidalis*, but not *E. bovis*, were able to undergo asexual sporogony after a month at − 18 °C (Lassen and Seppä-Lassila 2014). Thus, in cases of continuing bovine coccidiosis outbreaks, the management of the herd as well as species identification should be critically assessed, particularly with respect to hygiene, feeding, animal density, and floor types to achieve a significant reduction of infective oocysts in stables and premises. A quick method to assess the effects of novel disinfectants or anti-coccidial drug treatments on *Eimeria* sporogony could be a helpful tool to manage cattle coccidiosis in the future and/or to analyze in detail effectiveness of applied environmental disinfectant treatments on sporogony.

As already stated, structural properties of *Eimeria* oocysts help them survive long periods of time within harsh environments and which have been associated to the oocyst wall composed of two layers (Ferguson et al. 2003; Mai et al. 2009). The oocyst wall of ruminant *Eimeria* is composed of two distinct layers: the outer layer (500–200 nm) and the inner layer (40 nm) which prevent mechanical as well as chemical damage of sporozoites. This is one of the reasons why breaking ruminant *Eimeria* oocysts under laboratory conditions requires special protocols including use of mechanical disruptive proceedings (e.g., high-speed shaking with glass beads) or enzymatic digestion to disrupt the wall (Nyberg and Hammond 1965; Kowalik and Zahner 1999; Hermosilla et al. 2002). In fact, cleaning of oocysts with bleach, or storage in harsh oxidant medium (i.e., potassium dichromate), showed to not alter their infectivity (Hermosilla et al. 2002). The wall is also impermeable to many water-soluble disinfectants and detergents (Monné and Hönig 1954). Furthermore, this resistant wall structure has clearly interfered with application of molecular tools to analyze intra-oocyst structures in detail, including immunofluorescence, confocal, or electron microscopy analysis. Given that, commonly used fluorescent dyes do not penetrate highly resistant *Eimeria* oocyst wall and might alter sporogony process. This fact might explain why most ultrastructural aspects and/or fluorescence analyses of bovine *Eimeria* species have focused so far on endogenous merogony and gamogony stages (Taubert et al. 2008) but with exception of exogenous sporogony (Lassen and Seppä-Lassila 2014).

Therefore, the aim of this study was to investigate for the first time aerobic-dependent sporogony of *E. bovis* oocysts to be as close as possible to an in vivo situation by using the novel 3D live cell holotomography microscopy based on refractive indexes (RI) of intracellular structures without requiring membrane rupture or oocyst fixation to identify intra-oocyst organelles. The 3D holotomography imaging was performed on external and internal *E. bovis* oocyst changes during the sporogony process. This novel technique allowed detection of several sporogony phases sub-divided into first, second, and third sporogony phases. Besides, based on the high quality of 3D live cell holotomography images, quantification of metabolic dry material of sporont, sporoblast, sporocyst, and sporozoite stages was possible to achieve, allowing fast and easy discriminating between different sporulation phases. These new data will contribute as a baseline study on exogenous sporogony permitting better understanding of different cytokinetic and metabolic pathways to be involved in *E. bovis* sporogony, as well as a rapid tool to identify or assess the sporulation degree after exposure to novel disinfectants/drugs or harsh environmental conditions.

## Material and methods

### Ethics

All animal procedures were performed according to the Justus Liebig University Giessen Animal Care Committee guidelines, approved by the Ethic Commission for Experimental Animal Studies of the State of Hesse (Regierungspräsidium Giessen, GI 18/ 10 No A37/2011, JLU Giessen-No. 494) and in accordance to current German Animal Protection Laws.

### Parasites

For parasite propagation, two 8-week-old Holstein Friesian calves were kept in autoclaved metabolic cages within a large animal facility unit of the Institute of Parasitology (JLU Giessen), equipped with a laminar flow entrance and restricted access to avoid any bovine *Eimeria* spp. exposure. After deemed, parasite-free calves were orally infected with 3 × 10^4^ sporulated *E. bovis* oocysts (strain H) suspended in tap water according to Hermosilla et al. (2002). During patency, non-sporulated oocysts were isolated from feces beginning at 18 days p.i. according to Jackson (Jackson 1964). Feces containing oocysts were washed sequentially through a set of three sieves (pore sizes 850, 250, and 80 µm, respectively, Retsch®) with tap water. Final suspension was left to sediment overnight, and supernatant was discarded. The sediment was mixed 1:1 with saturated sucrose solution (1.3 g/mL) to a final density of 1.15 g/mL. The suspension was transferred into plastic trays (30 × 20 × 5 cm), and the level was horizontally adjusted. Plastic trays were filled to the top and thereafter covered with clean glass plates allowing complete contact of suspension with glass surfaces. Every 4 h, the glasses were carefully removed, and adherent oocysts were washed off with tap water into a container. The remaining suspension in plastic trays was stirred up, and the process was repeated up to six times or until few oocysts were isolated (microscopic examination, less than 5 oocysts per field of view at × 20 magnification). Collected *E. bovis* oocysts were diluted in tap water (1:1) and then centrifuged at 600 × *g* for 10 min.

### Exogenous Eimeria bovis sporogony

Isolated *E. bovis* un-sporulated oocysts were re-suspended in 2% potassium dichromate (K_2_Cr_2_O_7_; Merck) solution at room temperature (RT) with constant oxygenation as reported elsewhere (Duszynski and Wilber 1997). Every day, fifty oocysts were examined by light microscopy (inverted microscope IX81®, Olympus) to observe changes of bi-layered oocyst wall, sporont, sporoblast, Stieda body, and micropyle, as well as sporocysts containing complete sporozoite development. These oocyst structures were used as a guide to evaluate the sporulation process according to previous reports (Berto et al. 2014; Florião et al. 2016).

### 3D holotomographic live cell imaging of Eimeria bovis oocysts

Oocysts of *E. bovis* at different sporogony stages were carefully collected using a micropipette and washed twice with distilled water. Isolated oocysts were seeded into a 35-mm imaging dish plate (IBIDI®, Martinsried, Germany) with sterile phosphate buffered saline PBS pH 7.4, and the dish was placed inside a top-stage incubator (IBIDI®, Martinsried, Germany). Refractive index (RI)-based 3D holotomographic images were obtained by using a 3D Cell Explorer-Fluo (Nanolive, Switzerland) microscope equipped with a × 60 magnification (*λ* = 520 nm, sample exposure 0.2 mW/mm^2^) and a field depth of 30 µm. Images were captured and analyzed using STEVE® software (Nanolive) to obtain a RI-based z-stack. Several oocysts were acquired in the same field of view, but images were further analyzed one by one to identify the different sporulation stages. Images were composed of 96 slices, but only those whose whole oocyst structure was captured were chosen for further analysis and final pictures. All slices chosen are displayed as maximum z-projections, and gamma, brightness, and contrast were adjusted (identically for compared image sets) using Fiji® software (Schindelin et al. 2012).

### Statistical analysis

All presented data were expressed as mean ± SEM. Data were analyzed by Kruskal–Wallis using GraphPad Prism®9 software. Differences between conditions were estimated by a non-parametric one-way ANOVA multiple comparison test, applying a significance level of 5% (*α* ≤ 0.05).

## Results

### Eimeria bovis sporogony results in six internal oocyst structures before sporozoite release

From the total amount of 50 oocysts, 40.3 ± 2.62 *E. bovis* oocysts completed sporogony (i.e., containing four sporocysts each of them with two fully developed sporozoites) within 6 days in 2% potassium dichromate incubation at RT. *E. bovis* oocysts with delayed sporogony needed approximately 9–10 days to reach mature sporocyst formation. Percentages of oocysts without sporogony were less than 10%. In order to identify whether *E. bovis*–sporulated oocysts contained vital sporozoites, oocysts were submitted to an excystation protocol in vitro. Free-released sporozoites were then used to assess host cell invasion and further merogony development in primary bovine umbilical vein endothelial cells (BUVEC) in vitro. As such, extracellular sporozoite gliding motility, sporozoite host cell invasion, and trophozoite and macromeront formation with production of viable merozoites I were evaluated (Hermosilla et al. 2002, 2015; López-Osorio et al. 2020).

The in vitro sporogony was evaluated by a new 3D holotomography microscopy technology, in order to identify in detail membrane composition and cytoplasmic contents of diverse organelles without fixation or staining steps. In order to avoid background noise from medium detritus, the samples were washed in PBS before seeding them in a 35-mm imaging dish with a polymer coverslip bottom for high-end microscopy analysis.

For initial analysis, each image was acquired containing 90 slices, and then only the slices that contain the whole oocyst were selected and processed by a maximum z-projection algorithm. This process allowed us to identify the oocyst structure, inner volume, and dry mass of intracellular structures. The 3D rendering permits us to identify in detail the RI density of intra-oocyst structures. Our results show that the intracellular structures are better observed by using the RI density processing than the digital staining method available in the software. The acquisitions show a mix of different oocyst structures and maturation degrees suggesting an unsynchronized sporulation process. The analysis of examined oocysts unveils granules and/or extracellular vesicles contained within the circumplasm, the inner and outer oocyst membranes, the micropyle, the polar granule, the sporont, the sporoblast, the sporocyst membrane, and the fully developed sporozoites containing marked refractile bodies.

**Fig. 1:**
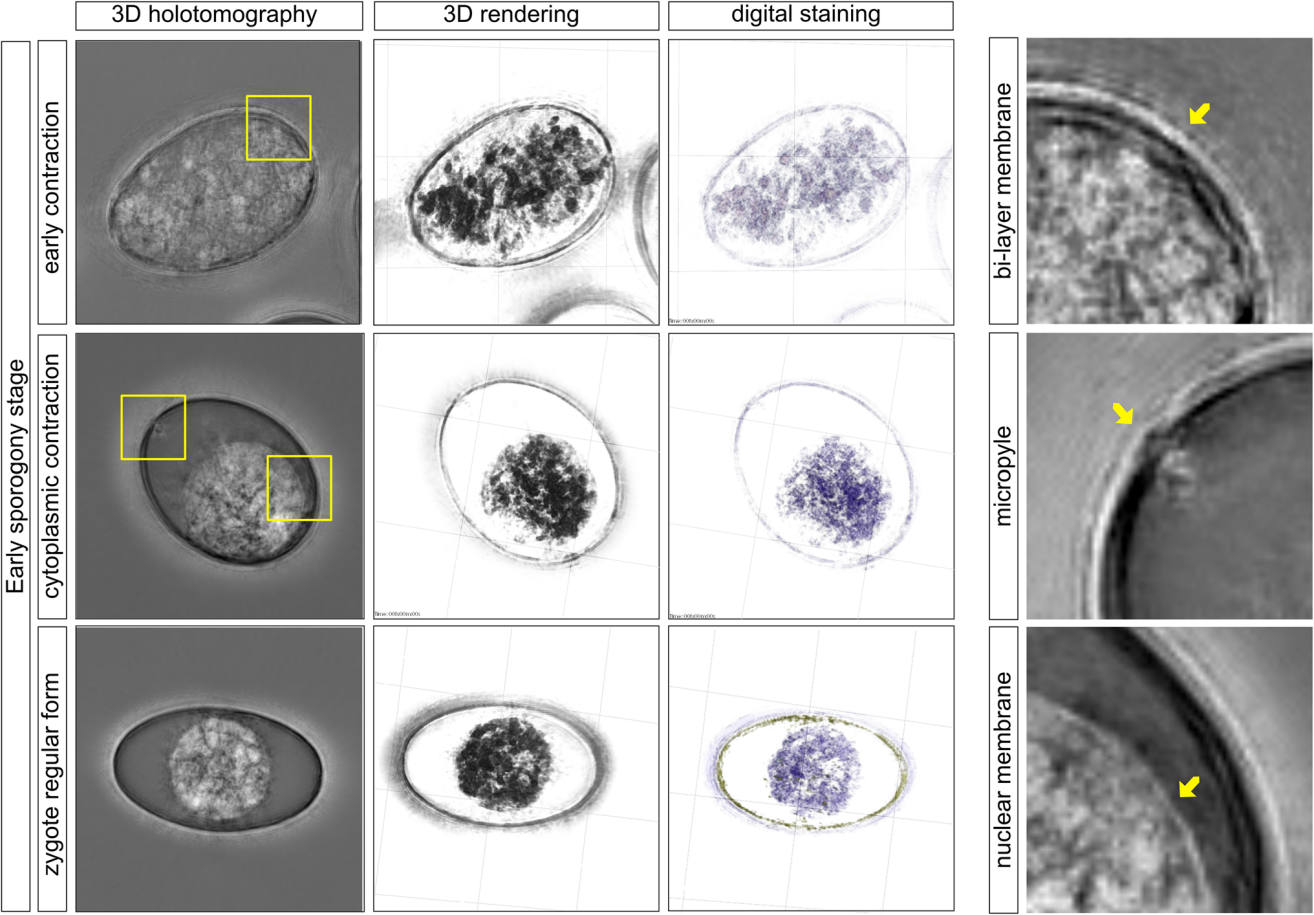
Early sporogony stage of *E. bovis.* First sporogony stage in *E. bovis* oocyst began with a diffuse zygote closely related with the inner oocyst wall (early contraction). Magnification of oocyst wall in contact with the zygote is shown in the 3D rendering image. The digital staining shows homogeneous distribution of the granules across the circumplasm (purple). After the cytoplasmic contraction, the zygote becomes rounded and placed in the center of the oocyst (regular form). The nucleus can be seen as a pale spot in the center of the mass. Closely related with the inner wall and the zygote, some vesicles were seen with a different refractive index (yellow). Images were analyzed using STEVE® software (Nanolive) to obtain a refractive index-based z-stack (3D holotomography), the rendering format, and the digital staining based on the refractory index

Freshly isolated *E. bovis* oocysts from feces had a large zygote being in close contact with the oocyst bi-layered wall containing several cytoplasmic granules homogeneously distributed in all un-sporulated oocysts (Fig. 1: early contraction phase). At this stage, both the inner and outer oocyst membranes became visible (Fig. 1, bi-layer membrane, yellow arrow). The first sporogonial stage began with the late cytoplasmic contraction phase (CCP), in which the cytoplasmic content is restricted to half volume of the oocyst (Fig. 1). At this early sporogony phase, the micropyle was identified as well as the nuclear membrane (Fig. 1, yellow arrows). The early sporogony stage (ESS) can be sub-divided into three phases, i.e., early CCP, late CCP, and the zygote regular form (ZRF) phases. The total time of ESS in *E. bovis* was between 24 and 48 h, and the late CCP occurred after oocysts have been suspended in 2% potassium dichromate (5 oocysts from 50 were sporulated). The zygote shapes changed from circular to oval, and the nucleus was located in a central position of oocysts undergoing sporogony, as a clear spot inside the cytoplasmic mass (Fig. 1, zygote regular form). The size of the sporont at this time point was 17.32 ± 0.59 µm in diameter (*n* = 10).

**Fig. 2:**
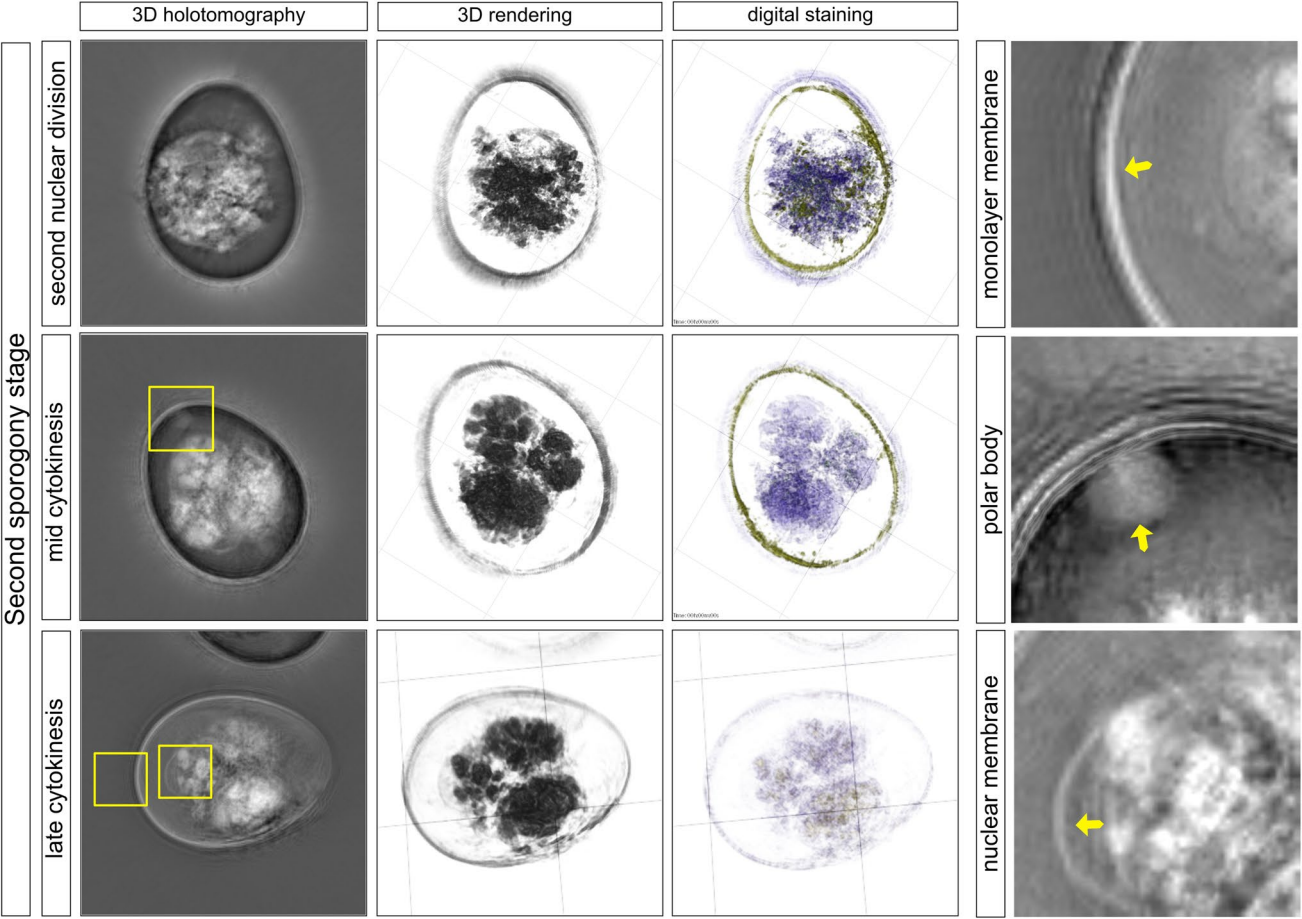
Second sporogony stage of *E. bovis.* The nuclear division took place in this stage. The pale spot is no longer in the center of the mass. The nucleus is divided twice, to form 4 nuclei, which can be located in the periphery of the mass. Closely related with the inner wall and the zygote, some vesicles were seen with a different refractive index (yellow square). Cytokinesis begins with the early formation of the four sporoblasts (early cytokinesis). Four protrusions, located in perpendicular directions, can be seen in the surface of the zygote (late cytokinesis). Images were analyzed using STEVE® software (Nanolive) to obtain a refractive index-based z-stack (3D holotomography), the rendering format, and the digital staining based on the refractory index

**Fig. 3:**
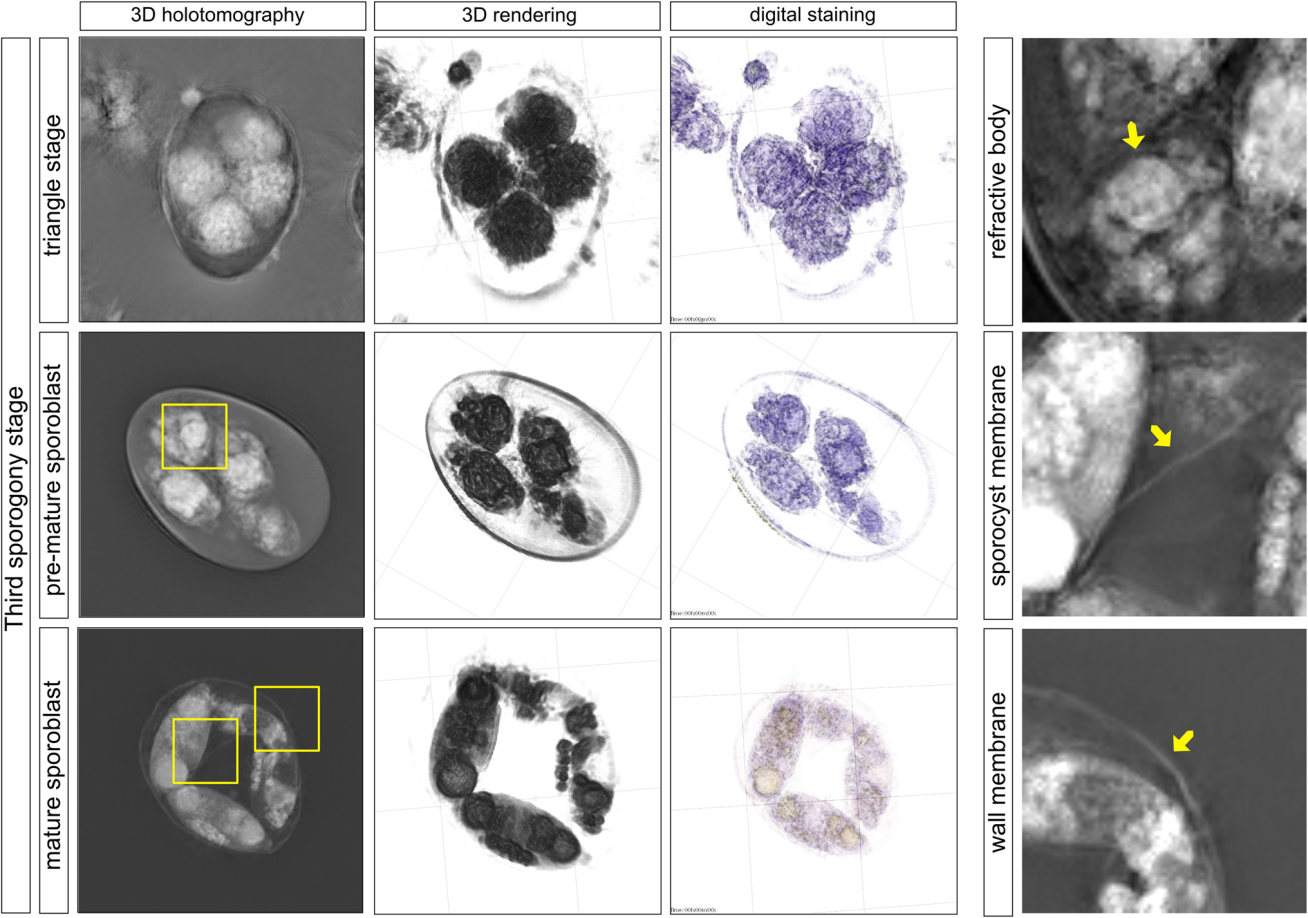
Late sporogony stage of *E. bovis.* The protrusions from the late cytokinesis become more prominent, increase in size, and become spherical, forming a clover-shaped structure, until they separate from each other forming the four sporoblasts. The sporoblast becomes elongated and forms a cigar-like shape. Each sporoblast contains granular mass and vacuoles, which develop into the residual body of the sporocyst. The sporoblast continued to become into an oval-shaped sporocyst. Here, a single division of the nuclei occurred, and then a division of the cytoplasm, which results in the formation of two sporozoites, each one with a visible big refractile body, and the residual body (light-refracting granules). Stieda bodies can be seen as a white tiny lid in one side of the sporocyst (mature oocyst). After the Stieda body and refractile body of the sporozoites are visible, the oocyst is considered mature. Images were analyzed using STEVE® software (Nanolive) to obtain a refractive index-based z-stack (3D holotomography), the rendering format, and the digital staining based on the refractory index

The second phase of *E. bovis* sporogony displayed three main stages, i.e., the second nuclear division (SND) and the mid- and late cytokinesis. The SND was only observed 48 h post incubation in potassium dichromate solution. The SND phase was completed at 72 h post incubation (Fig. 2). The SND was observed as a cytoplasmic decompression, and the nuclear membrane was practically absent (Fig. 2, SND). Alongside, the oocyst shape changed from an initial oval form into a circular form. During the second phase of *E. bovis* sporogony, the cytoplasm was gradually reduced, and three/four nuclei per oocysts surrounded by a thin membrane became visible (Fig. 2, yellow arrow in a nuclear membrane image). The bi-layer wall seemed to be fused in some parts of analyzed oocysts and has been observed as a single membrane (Fig. 2, yellow arrow in monolayer membrane). Moreover, at this point, a balloon-like structure was unveiled at the apical zone of oocysts, which corresponded well to the polar granule localization. In this process, *E. bovis* sporonts measured 21 × 18.9 µm (*n* = 10) and were gradually transformed into 4 sporoblasts (9.95 × 7.88 µm; *n* = 10) by progressive constriction of their bodies until cleavage was completed (Fig. 3, late cytokinesis). The summary of the total number of oocysts that we recorded in each sporulation step is shown in Table 1.

**Table 1:** *E. bovis* sporogony stage quantification

Sporogony* stages					
Early		Second		Third	
Stage	Number	Stage	Number	Stage	Number
Zygote regular form	18	Late cytokinesis	2	Mature sporoblast	2
Cytoplasmic contraction	6	Mid cytokinesis	5	Pre-mature sporoblast	2
Early contraction	7	Second nuclear division	6	Triangle stage	2

^*^Fifty oocysts were counted and classified according to the different sporogony stages they were

**Fig. 4:**
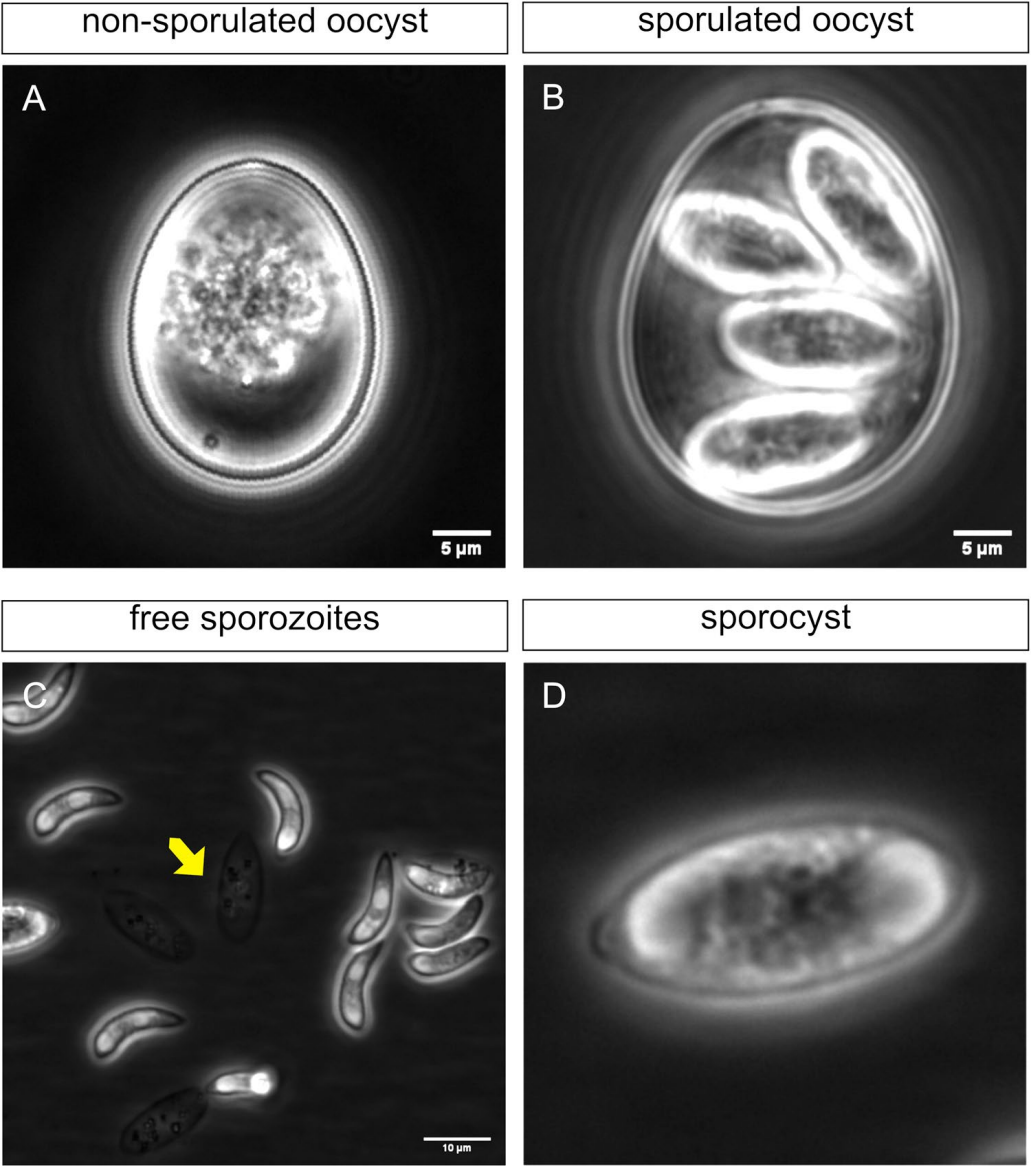
*Eimeria bovis* oocyst. Non-sporulated (**A**) and sporulated (**B**) oocysts. The four sporocysts of *E. bovis* sporulated oocysts are ovoid and contain two sporozoites. The average length was 14.7 ± 2.22 µm and width 6.5 ± 0.58. Normally, the length and width of the sporocyst are one-fourth and one to half of the oocyst. Each sporozoite showed a big refractile body in the base opposite to their partner. **C** Free sporozoites and residual material inside the sporocyst were clearly visible as a cumulus of extracellular vesicles (yellow arrow). The Stieda bodies were located in the narrowed end of the sporocyst. These are plugs which are covering the sporocyst micropyle. **D** Sporocyst

The third sporogony stage runs with three main steps, triangle, pre-mature, and mature sporoblast stages. Here, the four nucleus divisions became evident as well as first development of two immature sporozoites enclosed within sporoblasts (Fig. 3). Newly formed sporoblasts became rounded and obtained a clover-like shape. After this, sporoblasts began to elongate forming the cigar-shaped sporoblasts (12.78 ± 1.3 × 6.19 ± 0.72 µm). During the third sporogony stage, also refractive bodies (RF) of sporozoites became visible for the first time (Fig. 3, yellow arrow). After RF formation, the Stieda body and previously formed RF were visible in newly formed sporozoite stages within four sporocysts (Fig. 4B). Sporocysts were considered fully mature at 96–120 h of incubation containing two well-developed sporozoites with clear anterior and posterior RF formation. At the end of sporogony (final sporogonial phase), residual material inside sporocysts (14.7 ± 2.22 × 6.5 ± 0.58 µm) was clearly visible as a cumulus of extracellular vesicles (Fig. 4C, yellow arrow). The same holds true for the oocyst circumplasm containing as well residual material collected in multiple granules or vesicles of different sizes. The sporocysts of *E. bovis* are ovoid and contain two sporozoites (Fig. 4D). Each sporozoite showed a large RF in the base opposite to their partner. The Stieda bodies (SB) were located at the narrow end of sporocysts. These SB were visible as plugs covering the sporocyst micropyle. This area represents the egress for sporozoites from sporocysts. During *E. bovis* excystation, circumplasmic granules were released in a fast-sorted way from opened micropyle. Interestingly, circumplasmic granules often surrounded free-released and highly motile sporozoites and kept in close contact to the sporozoite surface, while these infective stages were moving away from empty oocysts.

### Morphometric analysis of E. bovis oocyst structures as a useful tool for sporogony stage discrimination

**Fig. 5:**
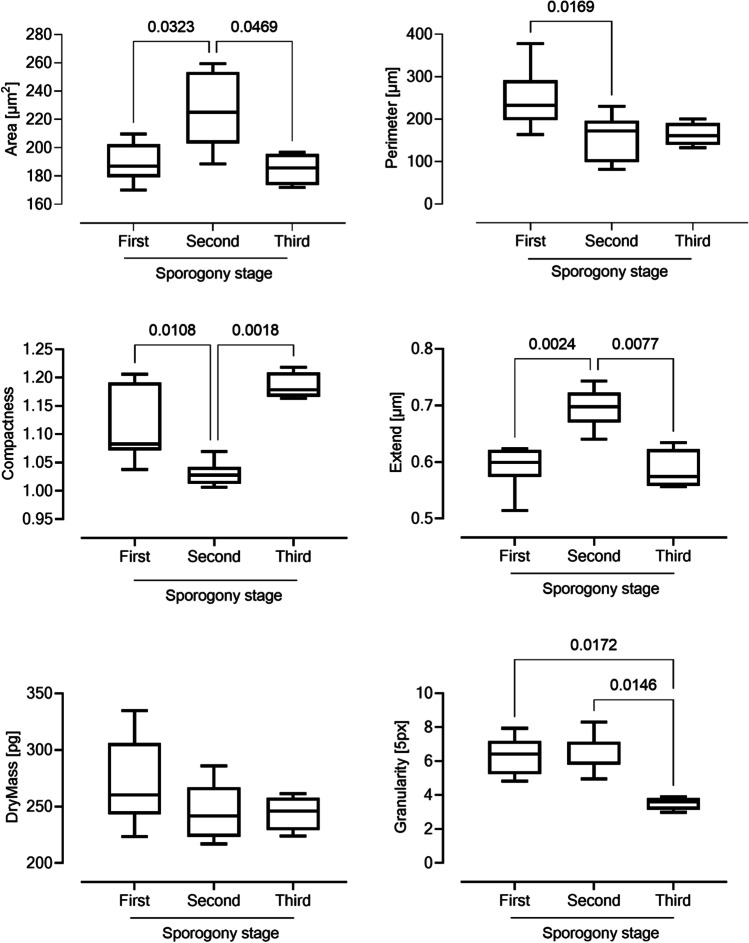
Morphometric characterization of the different stages observed during *E. bovis* sporulation using 3D microscopy. All oocyst stages recorded analyzed based on their RI in order to extract the area, perimeter, dry mass, compactness, extent, and granularity in each frame of the time-lapse experiment. The results show changes in the area, perimeter, compactness, extent, and granularity. The dry mass was decreased with time of maturation. Values are expressed as mean ± SEM. The value of *p* is informed in each graph

*Eimeria* spp*.* oocyst sporulation in the environment is conditioned by many factors including humidity, oxygen pressure, or temperature, which all can hamper production of infective units, namely, sporulated oocysts. However, the degree of oocyst sporulation is quite difficult to achieve in vitro. 3D holotomography microscopy analysis could be a powerful tool to evaluate this process in order to assess, for example, the effectiveness of a treatment against this infective stage. By using 3D-generated images, all sporulation stages were clearly identified, and by using additional image analysis tools, we measured several oocyst parameters. A CellProfiler pipeline was designed to load each 2D RI map; segment the contained oocysts using the primary objects detection module; and extract area, shape, and intensity features using the measurements modules. A critical point for proper oocyst detection was to use a correct threshold value, while automatic threshold detection is suited for most fluorescent microscopy images, whose signal dynamics are related to a different type of factors. In contrast, the RI images are quantitative and depend only on the nature of the biological object that is examined. Therefore, the threshold can be entered as a fixed value, ensuring the same analytical conditions in all the samples. The oocyst sizes were considered to encompass the full spectrum of potential diameters. Once segmented, these objects were finally used to extract the area, perimeter, dry mass, compactness, extent, and granularity in each frame of the time-lapse experiment. The data were exported as a*.csv* file. The results showed that the area of the oocyst was dramatically increased in the second sporogony stage, but it was not different between the first and third stages (Fig. 5). Nevertheless, the oocyst perimeter decreased according to the sporogony stages proceeded (Fig. 5). Two parameters that combined allowed a good discrimination of sporogony degree were compactness and dry mass, which were significantly increased and decreased when oocysts reached the last sporogony phase (Fig. 5). Finally, results on granularity of *E. bovis* oocysts clearly showed that oocysts in the third sporogonial stage displayed a reduction in granularity when compared with the previous two sporogonial phases.

## Discussion

Complete life cycle of *E. bovis* has been well documented in the past except for the exogenous sporogony generation (Hammond et al. 1946; Fayer and Hammond 1967). Asexual *E. bovis* oocyst sporogony is tightly regulated and complex and represents a necessary step for becoming infective stages, namely, a sporulated oocyst. Although these facts, *E. bovis* sporogony, as well as other ruminant species, has infrequently been explored in the past. To our best knowledge, this work represents first documentation of whole exogenous *E. bovis* sporogony using a novel live cell imaging 3D holotomographic microscopy analysis in vivo without fixation or interference through staining procedures*.*

Exogenous sporogony is an oxygen-dependent metabolic dividing process, and consequently Senger demonstrated that at least an oxygen tension of 15 mm of Hg was necessary for completing this parasitic replication (Senger 1959). Additionally, *Eimeria* oocysts also require suitable temperatures and relative humidity for fulfilling exogenous sporogony. Previous reports described complete *E. bovis* sporogony naturally occurring within 2–3 days under optimal temperature, oxygen, and humidity conditions, but in our study, *E. bovis* sporogony showed to be longer by 2–3 days. In the present study, 90% of oocyst sporulation rates were achieved after 6 days at constant RT (24–25 °C) and oxygenation, whereas in a former study, *E. bovis* oocysts were kept at constant 28 °C and needing 3 days for 90% sporulation (Pyziel and Demiaszkiewicz 2015). These results are consistent with observations of many authors with respect to the influence of temperature on *Eimeria* spp. sporogony. Just by one degree Celsius lower, namely 23 °C, under natural conditions, *E. bovis* sporogony was delayed by up to 25 days (Wagenbach and Burns 1969), and at much lower temperatures (3–5 °C) sporogony was prolonged for up to100 days demonstrating temperature-derived effects on exogenous *Eimeria* asexual replication (Pyziel and Demiaszkiewicz 2015). Most authors agreed that the use of anti-bacterial or anti-fungal agents (i.e., potassium dichromate) as oocyst conservation solution helped achieve higher sporogony rates under laboratory conditions (Lotze and Leek 1961; Graat et al. 1994).

Three main stages can be differentiated in *E. bovis* sporogony: (i) early sporogony stage (sub-divided in un-sporulated oocyst stage, less concentrated sporoblasts, and concentrated sporoblasts), (ii) mid sporogony stage (sub-divided in nuclear division and cytokinesis), and (iii) late sporogony stage (four rounded sporoblasts and mature sporoblasts). These sporogonial stages have been reported for other non-ruminant *Eimeria* species, such as chicken *E. maxima* and *E. tenella* (Wagenbach and Burns 1969). Nonetheless, for these two avian species, the classical pyramid-shaped sporoblasts were documented after 24 h of sporogony, which in the case of *E. bovis* oocysts were not seen. Instead, a clover-shaped sporoblast form was detected during aerobic *E. bovis* sporogony. Morphological differences of eimerian sporoblasts might result from the fact that *E. bovis* oocysts were kept in constant suspension conditions while performing asexual cellular division. Conversely, other sporogony-related studies used oocysts added to glass slides and covered with coverslips as performed in all avian *Eimeria* sporogony-related studies, which might have physically distorted forces on newly formed sporoblasts thereby shaping them differently from *E. bovis* sporoblasts. Interestingly, a similar sporoblast shape was previously described for Cystoisospora felis (former Isospora felis) sporogony. It shows two spherical sporoblast nuclei with central or peripheral localization, similar as we described here for *E. bovis* sporoblasts (Shah 1970).

After being excreted, the zygote cytoplasm starts to shrink within bi-layered *E. bovis* oocysts, thereby becoming a more spherical structure. It has been postulated that this zygote shrinkage is associated with oocyst dehydration. After oocyst dehydration, the remaining fluid between the zygote and the oocyst bi-layered wall, known as circumplasm, starts to contain small refracting bodies and bacteria (Yakimov et al. 1940). Exogenous oocyst dehydration has already been reported for a majority of coccidian parasites, with exception of the genera *Sarcocystis* and *Cryptosporidium*, both of which undergo endogenous sporogony within the host intestine under anaerobic conditions (Yakimov et al. 1940). In addition, some fish *Eimeria* species, i.e., *E. carpelli* and *E. subepithelialis*, are eliminated from infected host as fully sporulated oocysts (Yakimov et al. 1940).

The molecular content and precise function of granules in oocyst circumplasm are still unknown. During initial sporogony process of *E. bovis*, we detected via 3D holotomography analysis tiny structures and granules with different RI closely related to the oocyst wall and to the zygote components during sporulation. These spherical structures or granules then disappeared when zygote became a sporoblast. Structures with the same RI were found later on in free-released sporozoites surrounding their prominent apical and basal refractile bodies. More importantly, small granules/vesicles were additionally released while sporozoite egress from oocyst and occurring in a clearly sorted manner. These granules were in close contact to sporozoite surface and be found later on surrounding free-released and motile sporozoites. These circumplasm structures might represent functional extracellular vesicles (EV), microsomes, or exosomes which might influence sporozoite environment or modulate host cell innate immune reactions before cell invasion, but further investigation will be needed to clarify this assumption.

The awareness of *E. bovis* coccidiosis among farmers worldwide, particularly of dairy cattle, has significantly increased in the past decades, therefore raising further questions related to the control of highly resistant environmental oocysts and requesting disinfection strategies. Thus, we call for additional sporogony-related studies in order to gain new data on ruminant *Eimeria* spp. aerobic-dependent metabolic pathways leading to sufficient energy, blocking units and nutrients while achieving exogenous sporozoite development. Finally, the better understanding of metabolic pathways during aerobic *Eimeria* spp. sporogony might help identify new disinfection products or even drugs capable of interfering with this exogenous asexual parasite replication and impeding new animal infections.
